# Cost effective interventions for the prevention of cardiovascular disease in low and middle income countries: a systematic review

**DOI:** 10.1186/1471-2458-13-285

**Published:** 2013-03-28

**Authors:** Amir Shroufi, Rajiv Chowdhury, Raghupathy Anchala, Sarah Stevens, Patricia Blanco, Tha Han, Louis Niessen, Oscar H Franco

**Affiliations:** 1 Department of Public Health & Primary Care, Cardiovascular Epidemiology Unit, University of Cambridge, Strangeways Research Laboratory, Wort’s Causeway, Cambridge CB1 8RN, UK; 2 East of England Public Health and Social Care Directorate, Eastbrook, Shaftsbury Road, Cambridge CB2 8DF, UK; 3 Centre for Control of Chronic Diseases Bangladesh, icddrb, Dhaka, Bangladesh; 4 Johns Hopkins School of Public Health, Baltimore, USA; 5 University of East-Anglia, Norwich, UK; 6 Cardiovascular Epidemiology Group, Department of Epidemiology, Erasmus MC, Rotterdam, The Netherlands

## Abstract

**Background:**

While there is good evidence to show that behavioural and lifestyle interventions can reduce cardiovascular disease risk factors in affluent settings, less evidence exists in lower income settings.

This study systematically assesses the evidence on cost-effectiveness for preventive cardiovascular interventions in low and middle-income settings.

**Methods:**

Design: Systematic review of economic evaluations on interventions for prevention of cardiovascular disease.

Data sources: PubMed, Web of Knowledge, Scopus and Embase, Opensigle, the Cochrane database, Business Source Complete, the NHS Economic Evaluations Database, reference lists and email contact with experts.

Eligibility criteria for selecting studies: we included economic evaluations conducted in adults, reporting the effect of interventions to prevent cardiovascular disease in low and middle income countries as defined by the World Bank. The primary outcome was a change in cardiovascular disease occurrence including coronary heart disease, heart failure and stroke.

Data extraction: After selection of the studies, data were extracted by two independent investigators using a previously constructed tool and quality was evaluated using Drummond’s quality assessment score.

**Results:**

From 9731 search results we found 16 studies, which presented economic outcomes for interventions to prevent cardiovascular disease in low and middle income settings, with most of these reporting positive cost effectiveness results.

When the same interventions were evaluated across settings, within and between papers, the likelihood of an intervention being judged cost effective was generally lower in regions with lowest gross national income. While population based interventions were in most cases more cost effective, cost effectiveness estimates for individual pharmacological interventions were overall based upon a stronger evidence base.

**Conclusions:**

While more studies of cardiovascular preventive interventions are needed in low and mid income settings, the available high-level of evidence supports a wide range of interventions for the prevention of cardiovascular disease as being cost effective across all world regions.

## Background

Chronic diseases were estimated to account for approximately 50% of the total disease burden in low and middle-income countries in 2005 with further marked increases expected in the coming years [1].

It has been shown that the concomitant modification of multiple known risk factors (principally blood pressure and serum cholesterol concentration) could reduce cardiovascular disease to a large extent [2].

Both pharmacological and non-pharmacological strategies are likely to have a key role in tackling Cardiovascular Disease (CVD) in low and middle income countries [3]; non pharmacological strategies because of their potential for wide dissemination as well as their ability to be delivered more cheaply than pharmacological strategies to low and middle income populations, [4-6] pharmacological strategies because of the large absolute benefits conferred to those treated and the greater certainty in attribution of benefits [7].

While there is evidence to show that population-based and lifestyle interventions can reduce cardiovascular disease risk factors in affluent settings [8], as well as some evidence supportive of longer-term benefits in disease reduction, [6] less evidence exists in lower income settings. To generalise results from high income setting is not entirely satisfactory because reasonable thresholds for cost effectiveness will vary markedly – as will affordability [9]. Additionally setting specific information is important because population-based and preventive interventions are often, to some extent, context specific.

In this paper we evaluate and summarise the existing evidence on the cost-effectiveness of interventions for the prevention (primary and secondary) of cardiovascular disease in low and middle income countries. Furthermore, we describe how the level of cost-effectiveness differs by setting and intervention type.

## Methods

### Eligibility criteria

Studies were included if they were [i] randomised controlled trials assessing any cardio-protective intervention to prevent fatal or non-fatal CVD events (including myocardial infarction, coronary heart disease, stroke and heart failure); [ii] cohort, case–control, cross sectional studies or controlled trials reporting economic outcomes, or studies utilising the results of such studies to model economic outcomes; [iii] reported economic outcomes in terms of costs per YLG (years of life gained)/events averted, or cost-utility ratios, (ie, cost per QALY (quality adjusted life year) or DALY (disability adjusted life year)) of interventions aimed to prevent CVD; [iv] included adult participants (≥ 18 years old); and [v] published in any language. We excluded studies if they [vi] were letters, abstracts, case reports, editorials, descriptive studies, ecological studies or conference proceedings; [vii] involved non-human subjects; [viii] were conducted in affluent settings/rich countries (see Additional file 1: Appendix 1); [ix] provided only participant reported outcomes and [x] assessed only the effect of surgical interventions.

### Information sources

Between 1st November 2010 and 17 January 2011 (date last searched) we comprehensively searched the electronic databases Pubmed, Web of Knowledge, Scopus, Embase, Opensigle, the Cochrane database, Business Source Complete and the NHS Economic Evaluations Database. We also carried out domain-limited World Wide Web searches. (who.org + .htai.org + inahta.org) No limits were placed on the language or year of publication. Once articles for full text review were identified their references were checked for additional relevant publications. We also contacted their authors directly requesting relevant additional information and details of any related unpublished studies.

### Search strategy

Our full Pub-Med search strategy is shown below. This was translated for use in other databases with the help of an experienced librarian.

(((((((((((low and middle income countr*[Title/Abstract]) OR low income countr*[Title/Abstract]) OR Low OR middle income countr*[Title/Abstract]) OR LMIC[Title/Abstract]) OR developing countr*[Title/Abstract]) OR high income countr*[Title/Abstract]) OR undeveloped countr*[Title/Abstract]) OR south* AND asia[Title/Abstract]) OR middle income countr*[Title/Abstract]) OR resource limited[Title/Abstract])) OR (((((((((("Developing Countries"[Mesh] OR "Africa"[Mesh]) OR "Asia, Southeastern"[Mesh]) OR "Pacific Islands"[Mesh]) OR "Micronesia"[Mesh]) OR "Europe, Eastern"[Mesh]) OR "Middle East"[Mesh]) OR "Asia"[Mesh]) OR "Asia"[Mesh]) OR "Central America"[Mesh]) OR "South America"[Mesh])

AND

((((((((((((("Cardiovascular Diseases"[Mesh] OR "Coronary Artery Disease"[Mesh]) OR "Atherosclerosis"[Mesh]) OR "Coronary Disease"[Mesh]) OR "Myocardial Infarction"[Mesh]) OR "Myocardial Ischemia"[Mesh]) OR "Stroke"[Mesh]) OR myocardial[Title/Abstract]) OR ischaemic heart disease[Title/Abstract]) OR ischemic heart disease[Title/Abstract]) OR stroke[Title/Abstract]) OR brain vascular accident[Title/Abstract]) OR cerebrovascular[Title/Abstract]) OR cerebrovascular accident*[Title/Abstract]) OR CVA[Title/Abstract]

AND

((((((((taxation[Title/Abstract]) OR advertising[Title/Abstract]) OR social marketing[Title/Abstract])) OR ("Taxes"[Mesh] OR "Advertising as Topic"[Mesh])) OR (((((((((((((((((((diet* AND modification*[Title/Abstract]) OR salt[Title/Abstract]) OR sodium[Title/Abstract]) OR NaCl[Title/Abstract]) OR salt reduction[Title/Abstract]) OR smoking interventions[Title/Abstract]) OR exercise interventions[Title/Abstract]) OR physical activity[Title/Abstract]) OR multiple lifestyle[Title/Abstract]) OR dietary interventions[Title/Abstract]) OR diet[Title/Abstract]) OR food[Title/Abstract]) OR brief advice[Title/Abstract]) OR counselling[Title/Abstract]) OR incentive based[Title/Abstract]) OR active and passive[Title/Abstract]) OR dietary advice[Title/Abstract]) OR home health education[Title/Abstract]) OR lifestyle[Title/Abstract])) OR (((((((((((("Primary Prevention"[Mesh] OR "Secondary Prevention"[Mesh]) OR "prevention and control "[Subheading]) OR "Self Efficacy"[Mesh]) OR "Counseling"[Mesh]) OR "Directive Counseling"[Mesh]) OR "Disease Management"[Mesh]) OR "Behavior Control"[Mesh]) OR "Smoking Cessation"[Mesh]) OR "Behavior and Behavior Mechanisms"[Mesh]) OR "Sodium Chloride, Dietary"[Mesh]) OR "Feeding Behavior"[Mesh]) OR "Patient Education as Topic"[Mesh]))) OR (((((((((((((((((("Cardiovascular Agents"[Mesh] OR "Hydroxymethylglutaryl-CoA Reductase Inhibitors"[Mesh]) OR "Antihypertensive Agents"[Mesh]) OR "Aspirin"[Mesh]) OR "Angiotensin-Converting Enzyme Inhibitors"[Mesh]) OR "Calcium Channel Blockers"[Mesh]) OR "Adrenergic beta-Antagonists"[Mesh]) OR smoking cessation[Title/Abstract]) OR nicotine replacement[Title/Abstract]) OR bupropion[Title/Abstract]) OR bupropion[Title/Abstract]) OR varenicline[Title/Abstract]) OR chantix[Title/Abstract]) OR champix[Title/Abstract]) OR cytisine[Title/Abstract]) OR tabex[Title/Abstract]) OR clonidine[Title/Abstract]) OR nortriptyline[Title/Abstract]) OR nicorette[Title/Abstract])

AND

(((((((("Economics"[Mesh] OR "Socioeconomic Factors"[Mesh]) OR "Costs and Cost Analysis"[Mesh]) OR "Cost-Benefit Analysis"[Mesh]) OR cost effectiveness[Title/Abstract]) OR cost utility[Title/Abstract]) OR financ*[Title/Abstract]) OR economic[Title/Abstract]) OR monetary[Title/Abstract]) OR cost*[Title/Abstract]

### Study selection

Title and abstract for all studies identified by our search were screened by two independent reviewers (AS, RA, SS, TH, RC, PB, OhF) against our eligibility criteria to determine inclusion for full text review. AS reviewed all abstracts and RA, SS, TH, RC, PB and OhF equally shared the task of reviewing a duplicate list of all abstracts. Eligibility criteria were systematically applied to each abstract to derive a list for full text review, where an abstract was rejected the criteria barring eligibility were noted. In this way 2 lists of articles for further review were produced. These were then compared and disagreements were resolved by discussion; where disagreement persisted a third investigator was consulted (RA, OhF). In this way we arrived at an agreed list of articles for full text review.

All full text manuscripts were then assessed using a standardised checklist to ascertain definitively whether they met all eligibility criteria for this review. This was done independently by two reviewers (AS, OhF). Each reviewer then compared their selection with that of the other, reassessing against eligibility criteria in all cases of disagreement. Disagreements which persisted were resolved through discussion. Where agreement was not reached the opinion of a third party was sought (RA). The remaining studies were included within this review. For definitions used see Additional file 1: Appendix 1. For income groupings see Additional file 2: Appendix 2.

### Data collection process

From each study selected for inclusion we extracted a pre- specified set of data items using a data extraction form which was piloted before use. Data extraction was carried out by two independent reviewers (AS, OhF). The two reviewers compared data extraction results, resolving disagreements by discussion, before producing a final data extraction form which was entered into Epi Info.

### Data items

We extracted data on year of publication, study setting, geographic origin of publication, publication date, target population, intervention type, whether embedded within a trial, the nature analysis undertaken, modelling techniques used, main economic findings and funding source. We also extracted data on the analytic parameters used. To aid synthesis of results intervention effect estimates were categorised by metric used, the setting they related to and by intervention type.

### Risk of bias in individual studies (Quality review)

The quality of included articles was rated independently by 2 reviewers (AS, SS) according to the checklist for economic evaluations produced by Drummond [10]. We chose to present findings of quality review as either ++, +, or – as has been used by The UK National Institute for Health and Clinical Excellence (NICE) [11].

### Synthesis of results

We used broad categories of cost effectiveness to compare results between studies which we considered useful to facilitate comparison between studies while allowing for setting specific variation in costs and effects. Categories used are those suggested by WHO, whereby if cost/DALY ≤ (Gross national income) GNI per capita the intervention of interest would be considered: i) very cost effective, ii) with a cost/DALY of 1–3 times GNI per capita it would be categorised as cost effective and iii) with a cost/DALY more than 3 times GNI per capita classified as “not cost effective” [12]. We added a further category iv) of “extremely cost effective” in order to further differentiate cost effectiveness results. We arbitrarily defined this category as ¼ GNI per capita per DALY gained.

### Risk of bias across studies

We examined whether there was a systematic difference in the quality of evidence underpinning effectiveness estimates according to the modality of intervention. (pharmacological vs lifestyle interventions).

Study conducted and reported in line with the Preferred Reporting Items for Systematic Reviews and Meta-Analyses PRISMA statement guidelines [13].

## Results

### Study selection

Our initial search yielded 9729 results from all databases with two further studies obtained based upon responses of experts who were contacted. From these 9731 search results 93 studies were retrieved in full text after review of title and abstract against eligibility criteria. Review of the reference lists of the retrieved studies did not yield any additional studies.

**Figure 1:**
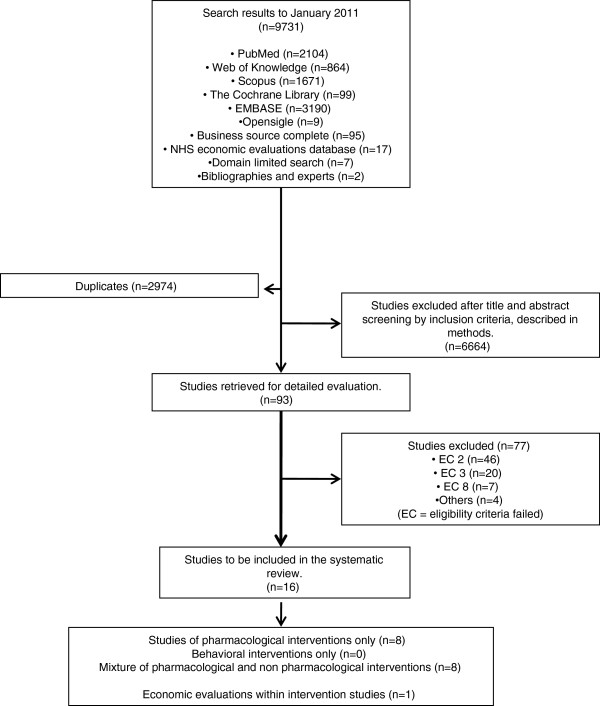
Flow diagram for the selection of studies evaluating the cost effectiveness of interventions for the prevention of cardiovascular disease in low and middle income countries: systematic review.

After review of the full text of these 93 studies a further 77 were excluded leaving 16 articles, which met our search criteria. In each case the criteria by which a study was deemed ineligible was recorded. Most studies were excluded at this stage because they were not of the study type required [14], or they did not provide results in terms of QALYs/DALYS/LYG. Eight studies were excluded due to being conducted in affluent settings (Figure 1).

### Study characteristics

The majority (12/16) of studies retrieved were published within 5 years of this review. Eight papers evaluated pharmacological interventions only, while a further 8 papers evaluated a mixture of both pharmacological and lifestyle interventions. None of the included papers evaluated only lifestyle interventions (Table 1).

**Table 1:** Summary of impacts assumed for interventions evaluated in retrieved studies and associated effect size estimates used to derive economic outcomes

	**Intervention**	**Different effects of intervention used**	**Effect estimates used within individual studies**	Source of effect estimate-trial type and principal* source
**Pharmacological interventions**	Polypill	Reduced absolute risk CVD	−20% [15]	Meta analysis [16]
		Reduced relative risk of CVD	RR=0.12 [17] for cardiovascular disease	Estimate based on RCT evidence [2]
			RR=0.29 for IHD and 0.4 for stroke (primary prevention) [18]	Overview of RCTs [19]
			RR=0.12 for CHD and RR=0.2 for stroke [20]	Multiplicative effects [21,22]
		Reduction in BP and cholesterol + reduced absolute risk (to account for effects of aspirin)	20% reduction in cholesterol+33% reduction in difference in BP between 115** and current + 20% reduction absolute risk CVD (to account for benefits aspirin) [23]	Product of estimates from RCT estimates used for Cholesterol and BP. For Aspirin [16]
			20% reduction in cholesterol+28% reduction in difference in BP between 115** and current + 18% reduction absolute risk CVD (to account for benefits aspirin) [24]	Product of estimates from RCT estimates used for Cholesterol and BP. For Aspirin [16]
	Treatment of “high“cholesterol	Reduction in total serum cholesterol concentration	−20% [15])	RCT [21]
			−20% [23]	RCT [21]
			−22% [25])	RCT [26]
		Reduction in relative risk of cardiovascular disease	RR=0.84 [20]	Heart Protection Study Group [21].
			RR=0.95 [17]	Meta analysis [27]
	Treatment of “high” BP	Reduction in relative risk of disease	RR=0.82 [17]	Overview of RCTs [28]
			RR=0.66 for stroke, RR=0.72 for CHD [20]	Overview of RCTS. [22]
		Reduction in the difference between SBP & 115 mmHg	−33% reduction [15]	Overview of RCTs [19]
			−33% reduction [23]	RCT [29]
		Blood pressure lowering	10 mmHg lowering of BP, yielding 40% RR reduction stroke and 14% reduction for coronary heart disease. [30].	Overview of randomised trials [19]
**Tobacco control**	Mass media smoking	Reduction in smoking prevalence	−2% [24])	Observational. Friend and Levy. 2002 [31].
			−1.5%[15]	Review of observational data [31]
	Price increase cigarettes	Reduction smoking attributable death	5-15% [32]	Review of observational data [33]
	Nicotine replacement therapy (gum)	Increased likelihood of cessation	OR=1.66 [34]	Systematic review [35]
		Increase in percentage using NRT who quit	5% [24]	
	Community pharmacist smoking cessation	Increase in proportion using cessation services who become long term quitters	14.3% continuous quit rate compared to 2.7 if usual care [36].	RCT [37]
	Bupropion-smoking cessation	Reduced relative risk of CVD	RR=0.8 [17]	Systematic review [38]
**Mass media interventions**	Mass media, diet/cholesterol	Reduced total serum cholesterol	−2% [15]	Cost effectiveness analysis [39]
			−2% [23]	Cost effectiveness analysis [39]
	Mass media salt/reductions food	Reduced total dietary salt intake	−20% (range 10-30%) [24]	Effect of salt on BP from meta analysis [40] Mass media effects not supported.
			−15% [15]	No reference for impact on salt intake, impact of salt reduction on BP from trial data [41]
		Reduced CVD prevalence	−4% [32]	Review [14] and expert opinion
	Combined mass media	Relative risk of CVD	RR=0.98 [17]	Meta analysis [42]
**Legislative Interventions**	Salt in bread-voluntary/other	Reduced CVD relative risk	RR=0.99 [17]	No reference for impact of legislation, review of trials supports impact of salt on CVD [43]
	Legislation on salt in food	Reduction in total dietary salt intake	30% reduction [23]	No reference for impact of legislation. Impact of salt on BP from observational data [44]
	Reduced salt intake via legislation + education	Reduced systolic BP	−2 mmHg (1-4) mmHg [32]	Review [14] and expert opinion
		Reduction in the difference between actual SBP & 115 mmHg	33% reduction [15]	

*Where multiple source citations provided the highest in hierarchy of evidence is shown.

**115 mmHg suggested as theoretical minimum risk level for systolic blood pressure by WHO.

Abbreviations: *CAD*, Coronary Artery Disease; *CVD*: Cardiovascular Disease; *SBP*, Systolic Blood pressure, *NRT*, Nicotine replacement Therapy, *WHO*, World Health Organisation.

(where available the source of effect estimates cited in each study has been shown).

In 6/16 cases the study originated from the setting of interest, with the other 10 studies originating from the USA or Europe. Within included papers over 20 different preventive interventions were evaluated in total. Geographical categories in which interventions were evaluated included World Bank and World Health Organisation (WHO) regions as well as individual country level.

Most studies (15/16) were based on stochastic simulation and we found only one economic evaluation embedded within an intervention study [45]. In 14/16 cases some form of sensitivity analysis was undertaken. In most cases (9/16) where this was carried out the impact of changes in the most consequential variable had a large (>1 order of magnitude) impact upon results.

2 studies declared pharmaceutical industry funding [46,47], 3 studies were government funded, [17,24,45] 7 were funded by another non-industry body [5,12,15,18,20,23,30] and in 4 cases funding source was not stated [25,34,36,48].

### Results of individual studies

All of the papers retrieved presented positive results, supporting some or all of the interventions considered as cost effective in the setting/s of interest. Where study authors categorised cost effectiveness using GNI the same thresholds as applied here were used. In all but one case [46] we arrived at the same categorisation as study authors.

#### Tobacco control

**Figure 2:**
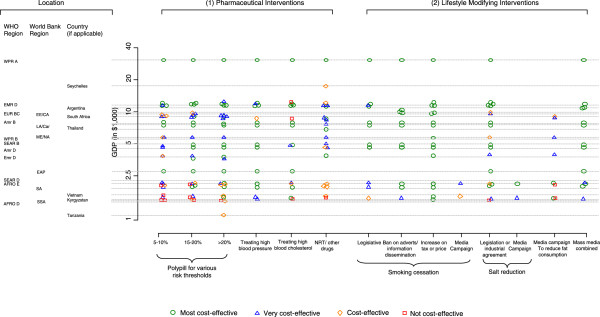
Levels of cost-effectiveness for different cardiovascular interventions in low and middle income countries arranged by annual gross domestic products (GDP).

We found 6 studies that evaluated tobacco control interventions in one or more low and middle income country [5,15,17,32,34,36]. Although personal interventions such as nicotine replacement therapy (NRT) were generally found to be cost effective, population-based interventions were much more cost effective (by an order of 10–100 fold) (see Figure 2).

#### Pharmacological primary prevention using an absolute risk based approach

7 studies evaluated the cost effectiveness of providing preventive medication on the basis of absolute risk. [15,17,18,20,23,24,30] 5 of these found this approach to be “very cost effective” or “cost effective” in all settings for which they evaluated this intervention, [17,18,23,24,30] with cost effectiveness generally increasing at higher risk thresholds for treatment (see Figure 2).

Less favourably, studies in Tanzania [20] and Kyrgyzstan [15] reported borderline and negative results respectively for the cost effectiveness of an absolute risk based approach.

#### Individual risk factor reduction approach

Of the 16 studies included, 5 evaluated the use of pharmaceuticals for individual risk factor lowering [15,23-25,30]. Drugs to lower “high” blood pressure were found to be in the “very cost effective” or “cost effective” range in all studies. In the case of statins, 3 studies reported them to be cost effective [23-25] while 2 studies found them to be not cost effective [15,17].

#### Pharmacological secondary prevention

All studies evaluating pharmacological secondary prevention of CVD found this approach to be in the “cost effective” or “very cost effective” range [15,46,47].

### Use of mass media

We found 5 studies, which evaluated the use of mass media to reduce salt consumption, to stop smoking, to improve dietary characteristics as well as joint campaigns [12,15,17,24,32].

The source of effect estimates underpinning cost effectiveness calculations were derived from a mixture of trial data, observational data and expert opinion. The use of mass media was generally found to be “very cost effective” (Figure 2) [12,15,17,24,32].

#### Other legislative interventions

Four of the studies included reported evaluations of legislative interventions such as the compulsory reduction of salt in food [12,17,23,32] (Figure 2). Interventions of this type were generally found to be very cost effective, or extremely cost effective, with the exception of salt lowering legislation in Sub Saharan Africa which was found not to be cost effective [32].

### Provenance of studies and study estimates of costs and effects

In all but one case [45] the population-based interventions estimates of effect size were derived from settings other than the setting of interest. Effect estimates were obtained utilising the results from a mixture of observational and experimental studies. We found variation in the nature of effects modelled for the same intervention as well as in the magnitude of change that intervention was assumed to produce (see Table 2).

**Table 2:** Summary of studies included

**Authors, year and title**	**Setting**	**Intervention/s**	**Main economic findings (outcome metric)**	**Target population**	**Quality score (Drummond)**	**In overall summary (Figure **2**)**
Huang Guangyong et al., 2000 [45] Cost effectiveness of the Beijing Fangshan cardiovascular prevention programme	China	Health education and anti-hypertensive drugs	Intervention found to be cost effective	Initially whole population, then high risk	+/−	No –limited comparability
Gaziano et al., 2007 [18] Cardiovascular disease prevention with a multidrug regimen in the developing world	6 World Bank Regions	Fixed dose combination therapy	Found cost effective in all world regions for primary and secondary prevention	Various	+ +	Yes
Caro et al. 1999 [25] International economic analysis of primary prevention of cardiovascular disease with Pravastatin in WOSCOPS	South Africa	Pravastatin for primary prevention	Authors describe Pravastatin as efficient for CVD primary prevention. Note, cost per LYG close to 3 X GNI per capita for study year. Thus cost/DALY likely to be > 3 X GNI/Capita	Men with high cholesterol	+	Yes
Rubinstein et al., 2009 [17] Generalised cost effectiveness analysis of a package of interventions to reduce cardiovascular disease in Buenos Aires, Argentina	Argentina	Personal pharmacological and non personal population-based interventions	All interventions cost effective with exception of statins to lower “high” cholesterol	Various	+ +	Yes
Anh Ha and Chisholm, 2010 [24] Cost effectiveness of intervention to prevent cardiovascular disease in Vietnam	Vietnam	Personal pharmacological and non personal population-based interventions.	Range of interventions judged cost effective and deliverable at low cost	Various	+ +	Yes
Gaziano et al., 2005 [30]. Cost effectiveness analysis of hypertension guidelines in South Africa	South Africa	Antihypertensive drugs	Absolute risk based initiation of therapy dominated a strategy of initiating medications based on blood pressure threshold alone	Hypertensive/high CVD risk.	+ +	Yes
Schuffham et al., 2006 [47]. The cost effectiveness of Fluvastatin in Hungary Following Successful PCI	Hungary	statins	Judged to be cost effective	Post PCI patients	+/−	No-limited generalisability
Gilbert et al., 2004 [34]. The cost effectiveness of pharmacological smoking cessation therapies in developing countries	Seychelles	Smoking cessation	Shown to be cost effective but affordability in LMIC settings questioned given high cost	Smokers	+	Yes
Robberstad et al., 2007 [20]. Cost effectiveness of medical interventions to prevent cardiovascular disease in a Sub-Saharan African country	Tanzania	Pharmaco-prevention including the polypill	Some interventions judged cost effective but affordability in this setting questioned	Those over age 45	+	Yes
Redekop et al., 2008 [46]. Costs and effects of secondary prevention with Perindopril in Stable Coronary Heart Disease in Poland	Poland	ACE inhibitos for secondary prevention	Authors report high probability for Perindopril effectiveness in secondary prevention. Using reported results against WHO criteria we find not cost effective – not study conclusions	Those with existing CHD	+/-	Yes
Thavorn et al., 2007 [36]. A cost effectiveness analysis of a community pharmacist-based smoking cessation programme in Thailand	Thailand	Nicotine replacement therapy	Authors find intervention to be cost saving. (cost/LYG)	Regular smokers	+	Yes
Araujo et al., 2007 [48]. Cost effectiveness and budget impact analysis of Rosuvastatin and Atorvastatin for LDL cholesterol and cardiovascular events lowering within the SUS scenario	Brazil	Branded statin	Rosuvasctatin found to be more cost effective than Atorvastatin	Those at high risk of CVD	-	No-comparison of 2 drugs of same class
Akkazieva et al., 2009 [15]. The health effects and costs of the interventions to control cardiovascular disease in Kyrgyzstan	Kyrgyzstan	Pharmacological and non personal population-based interventions	Wide range of cost effectiveness between interventions. Blood pressure lowering drugs and mass media most cost effective	Variable	+ +	Yes
Murray et al., 2003 [23]. Effectiveness and costs of interventions to lower systolic blood pressure and cholesterol	6 world bank regions	Pharmacological and non personal population-based interventions	Non personal interventions found to be most cost effective. Absolute risk based approach also found to be cost effective	Various	++	Yes
Disease Control Priorities Project * [32,49] Chapters 44: Prevention of Chronic Disease by Means of Diet and Lifestyle Changes. 45: Blood Pressure, Cholesterol and Bodyweight, 46: Tobacco Addiction.	6 world bank regions	Pharmacological and non personal population-based interventions.	Tobacco control interventions, salt reduction and multidrug therapy on the basis of absolute risk approach likely to be cost effective in most settings.	Various	++	Yes
WHO + Chisholm *[5,12] Comparative cost effectiveness of policy instruments for reducing the global burden of alcohol, tobacco and illicit drug use.	WHO regions	Personal and non personal interventions for tobacco control	Most interventions cost effective, non personal interventions such as taxation and legislation far more so than personal interventions such as NRT.	Smokers	++	Yes

Abbreviations: *CHD*: Coronary Heart Disease. *CVD*: Cardiovascular Disease. *GNI*: Gross National Income. *GDP*: Gross Domestic Product. *DALY*: Disability Adjusted Life Year. *QALY*: Quality Adjusted Life Year. *YLG*: Year of Life Gained. *NRT*: Nicotine Replacement Therapy. *LMIC*: Low and Middle Income. *ACE*: Angiotensin-Converting Enzyme. *LDL*: Low Density Lipoprotein. *PCI*: Percutaneous Coronary intervention. * Material concerning analysis presented in more than one journal article.

In the case of studies evaluating policy interventions, all effect estimates were based on the observed experience of other locations implementing such policies and/or expert opinion. Effects on risk factor levels were used to model expected changes in mortality.

Costs for population-based interventions were generally based upon a theoretical estimation of likely costs derived from summing individual strategy components, rather than by measuring the cost of delivering the intervention as a whole in a real life setting.

#### Parameters used in economic models

Most studies (11/16) used a 3% discount rate for costs and effects and all but one [46] used the same discount rate for costs and effects.

In the majority of papers retrieved (10/16) adherence was not incorporated directly in modelling, although by using trial data adherence was in effect incorporated at high levels in others. Where modelled, levels of adherence ranging from 50% [17] to 95% [15,17,24,25,47] were used.

Six studies used a lifetime time horizon, five used a 10 year horizon, one used 20 years and one 5 years with the remaining three studies not reporting the time horizon considered. Where altered in sensitivity analysis the potential impact of the chosen time horizon upon overall cost effectiveness was of at least one order of magnitude [45,46].

## Discussion

Economic evaluations of cardiovascular prevention in low and middle income countries have found a wide range of interventions to be cost effective across all world regions. Given the limited evidence base, findings should be interpreted with caution; yet can aid rational resource allocation and implementation.

Cost effectiveness estimates for pharmacological interventions were generally supported by stronger evidence than those for other interventions. Additionally agreement on effect sizes between studies was generally greater for pharmacological interventions.

Virtually none of the evaluations are fully based on data derived from LMICs. We found a consistent difference between the sources of the effect estimates for population-based interventions compared to personal interventions, with the latter generally based upon studies lower in the hierarchy of evidence (Table 2). The effectiveness estimates on personal, pharmacologic interventions for the most part are based on studies with reliable effect sizes in high income countries, supplemented with LMIC cost estimates. The evaluations of population-based interventions lack any RCT level of evidence which leads to greater uncertainties. Whereas individual interventions may be attractive, although based on HIC evidence, this is not the case in the area of tobacco control. Personal interventions such as Nicotine Replacement Therapy (NRT) appear to be far less cost effective than population-based interventions in low-resource settings. There are few trials of population-based interventions in low and middle income countries, leading to a lack of effectiveness information (Table 2).

Our findings are relevant for policy makers at the international level e.g. UN agencies with responsibilities in health across societal sectors, national government agencies and ministries, local private, non-profit and for-profit organizations in health care as well as professional medical societies and other health professional bodies. Given the uncertainties in the study findings, local relevance should be assessed, given disease epidemiology and available resources, and, next, taken into account, while making decisions and formulating country policies and guidelines.

Several methodological observations can be made. Most importantly, study methodologies across the identified articles are highly heterogeneous, in terms of analytic methods, input parameters and data used and the baseline against which the intervention of interest was evaluated. We utilise a null baseline to aid comparison, real world costs of implementing a given intervention may be higher if on going activities need to be wound down. Choice of time horizon was of particular consequence and some interventions were found on sensitivity analysis to move from cost ineffective as this parameter was altered [46,47]. The adoption of standard parameters for discounting rates, time horizons and study perspectives would help address this as would widespread adoption of a standard (counterfactual or null) baseline as proposed by WHO [12]. The published articles and background documentations do not allow for a detailed analysis of how this diversity would affect our comparison of the study findings nor do they allow adaptations of the calculations to facilitate a better comparison.

Next, most studies use known or predicted changes in risk factors, associated with each intervention of interest, within a stochastic or deterministic model, to estimate the anticipated changes in disease occurrence that would result. The Framingham equations, employed in most studies, also limit the reliability of results as they under predict risk in high risk populations while over predicting risk in low risk populations [50-53] Resulting cost effectiveness estimates may therefore be unduly favourable in low risk populations and vice versa. Lastly, most studies do not distinguish trial efficacy results from real-life effectiveness of implemented interventions. Especially, in many rural and urban areas, provider compliance, system compliance, and patient compliance, may lower the impact of the intervention and may raise the health care costs and broader societal costs for patients or the existing systems (see Additional file 3: Appendix 3).

Finally, the limitations of the cost effectiveness categorisation we have used should be acknowledged. Specifically, presently labelling an intervention as “cost effective” at less than 3 x GNI/capita does not necessarily imply that it should be adopted. Shifting resources from this to another intervention, even a very cost effective one, could lead to unacceptable transaction costs and be unwise if there are other compelling societal reasons to allocate resources in a different way.

## Conclusions

In sum, there is evidence supportive of a wide range of interventions to prevent cardiovascular disease in most parts of the world; nevertheless further setting specific research of preventive interventions in this field is needed and should include economic evaluation. Lifestyle interventions appear to be of generally greater cost effectiveness, while pharmacological interventions offer an impact of greater certainty and magnitude. These modalities of interventions can thus be seen as complementary, offsetting potential gain against certainty of outcome. Policymakers should aim to balance distribution of relevant resources between these areas, favouring the most cost effective in each class, while accounting for other criteria such as, affordability, access, and equity. Healthcare infrastructures concerned differ markedly [54], both among countries and within countries.

The economic evidence on both pharmacological and lifestyle interventions supports large-scale implementation strategies and efforts in all settings confronted with the growing NCD epidemic.

## Competing interests

The authors declare that they have no competing interest.

## Authors’ contribution

OhF and AS developed the idea for the study; OhF, LN, AS participated in the concept and design . RC, RA, SS, PB, TH; OhF and AS contributed to the systematic review process; RC produced the summary analysis presented as Figure 2. All authors contributed to the writing of this manuscript. All authors read and approved the final manuscript.

## Authors’ information

Rajiv Chowdhury and Raghupathy Anchala joint second author.

## Pre-publication history

The pre-publication history for this paper can be accessed here:

http://www.biomedcentral.com/1471-2458/13/285/prepub

## Supplementary Material

Additional file 1: Appendix 1 Definitions used in this review. Click here for file

Additional file 2: Appendix 2 Income groupings – WHO World Health Report 2008 (http://www.who.int/whosis/whostat/EN_WHS08_Full.pdf). Click here for file

Additional file 3: Appendix 3 Findings from studies reporting costs per treated individual. Per capita costs for selected interventions to prevent cardiovascular disease in LMIC settings compared to per capita expenditure on health for the year considered in that study. Click here for file
