# Supplementary material for: Cost effective interventions for the prevention of cardiovascular disease in low and middle income countries: a systematic review
Source: BMC Public Health. 2013 Mar 28;13:285. doi: 10.1186/1471-2458-13-285 (PMC3623661; doi:10.1186/1471-2458-13-285)
Supplement: Additional file 1: Appendix 1 — Definitions used in this review. [file 1471-2458-13-285-S1.doc]

Appendix 1 Income groupings – WHO World Health Report 2008 (http://www.who.int/whosis/whostat/EN_WHS08_Full.pdf)

**Low income:** Afghanistan, Bangladesh, Benin, Burkina Faso, Burundi, Cambodia, Central African Republic, Chad, Comoros, Côte d’Ivoire, Democratic People’s Republic of Korea, Democratic Republic of the Congo, Eritrea, Ethiopia, Gambia, Ghana, Guinea, Guinea-Bissau, Haiti, India, Kenya, Kyrgyzstan, Lao People’s Democratic Republic, Liberia, Madagascar, Malawi, Mali, Mauritania, Mongolia, Mozambique, Myanmar, Nepal, Niger, Nigeria, Pakistan, Papua New Guinea, Rwanda, Sao Tome and Principe, Senegal, Sierra Leone, Solomon Islands, Somalia, Sudan, Tajikistan, Timor-Leste, Togo, Uganda, United Republic of Tanzania, Uzbekistan, Viet Nam, Yemen, Zambia, Zimbabwe

**Lower middle income:** Albania, Algeria, Angola, Armenia, Azerbaijan, Belarus, Bhutan, Bolivia, Bosnia and Herzegovina, Cameroon, Cape Verde, China, Colombia, Congo, Cuba, Djibouti, Dominican Republic, Ecuador, Egypt, El Salvador, Fiji, Georgia, Guatemala, Guyana, Honduras, Indonesia, Iran (Islamic Republic of), Iraq, Jamaica, Jordan, Kiribati, Lesotho, Maldives, Marshall Islands, Micronesia (Federated States of), Morocco, Namibia, Nicaragua, Paraguay, Peru, Philippines, Republic of Moldova, Samoa, Sri Lanka, Suriname, Swaziland, Syrian Arab Republic, Thailand, The former Yugoslav Republic of Macedonia, Tonga, Tunisia, Turkmenistan, Ukraine, Vanuatu

**Upper middle income:** Argentina, Belize, Botswana, Brazil, Bulgaria, Chile, Costa Rica, Croatia, Dominica, Equatorial Guinea, Gabon, Grenada, Hungary, Kazakhstan, Latvia, Lebanon, Libyan Arab Jamahiriya, Lithuania, Malaysia, Mauritius, Mexico, Montenegro, Oman, Palau, Panama, Poland, Romania, Russian Federation, Saint Kitts and Nevis, Saint Lucia, Saint Vincent and the Grenadines, Serbia, Seychelles, Slovakia, South Africa, Turkey, Uruguay, Venezuela (Bolivarian Republic of)

**High income:** Andorra, Antigua and Barbuda, Australia, Austria, Bahamas, Bahrain, Barbados, Belgium, Brunei Darussalam, Canada, Cyprus, Czech Republic, Denmark, Estonia, Finland, France, Germany, Greece, Iceland, Ireland, Israel, Italy, Japan, Kuwait, Luxembourg, Malta, Monaco, Netherlands, New Zealand, Norway, Portugal, Qatar, Republic of Korea, San Marino, Saudi Arabia, Singapore, Slovenia, Spain, Sweden, Switzerland, Trinidad and Tobago, United Arab Emirates, United Kingdom, United States of America Cook Islands, Nauru, Niue and Tuvalu are not categorized into income groups and are therefore excluded from the computation of aggregate indices by income group.
