# Supplementary material for: Cost effective interventions for the prevention of cardiovascular disease in low and middle income countries: a systematic review
Source: BMC Public Health. 2013 Mar 28;13:285. doi: 10.1186/1471-2458-13-285 (PMC3623661; doi:10.1186/1471-2458-13-285)
Supplement: Additional file 2: Appendix 2 — Income groupings – WHO World Health Report 2008 (http://www.who.int/whosis/whostat/EN_WHS08_Full.pdf). [file 1471-2458-13-285-S2.doc]

Appendix 2: Findings from studies reporting costs per treated individual. Per capita costs for selected interventions to prevent cardiovascular disease in LMIC settings compared to per capita expenditure on health for the year considered in that study

| **Study author, year of publication and setting** | **Year for which results were modelled / base year** | **Intervention** | **Estimated cost per treated individual of intervention per year** | **Total health spend per capita per year**  **US$, Int$**  **(% which public)** | **% GDP spent on health care** |
| --- | --- | --- | --- | --- | --- |
| Duc Anh Ha et al 2010. (Vietnam) | 2007 | Mass media campaign | US$ 0.06/capita/yr | 58 US$ (2007)  183 Int $ (2007)  (39%) | 7% (2007) |
|  |  | Polypill for>5% risk | US$2.9/capita/year |  |  |
| Akkazieva et al. 2009  (Kyrgyzstan) | 2008 | Mass media salt campaign | 5 Som/year  (0.1 US$)** | 36 US$ (2007)*  120 Int$ (2007)*  (54%) | 7% (2007)* |
|  |  | Combination drug treatment (at 35% risk) | 480 Som/year  10 US$** |  |  |
| Caro et al.1999  (South Africa) | 1997 | Statins for high cholesterol | 896 US$/year  (1997rates) | 297 US$ (2003)*  608 int$(2003)*  (35%) | 8% (2003)* |
| Gilbert et al. 1999  (Seychelles) | 2003 | Nicotine patch | US$ 321 | 478 US$(2003)  879 int$(2003)  (77%) | 6% (2003) |
|  |  | Nicotine spray | US$ 1145 (2003 rates) |  |  |
| Robberstad et al. 2007  (Tanzania) | 2005 | Diuretics for high risk | US$85 | 14 US$(2005)  40 Int$(2005)  (49%) | 4% (2005) |
|  |  | Polypill | US$557 |  |  |

***closest year to study year for which data could be found**

**** using current exchange rates rather than the exchange rate for the study year**

**Where multiple interventions are considered in one study contrasting examples are provided**
