# Supplementary material for: Cost effective interventions for the prevention of cardiovascular disease in low and middle income countries: a systematic review
Source: BMC Public Health. 2013 Mar 28;13:285. doi: 10.1186/1471-2458-13-285 (PMC3623661; doi:10.1186/1471-2458-13-285)
Supplement: Additional file 3: Appendix 3 — Findings from studies reporting costs per treated individual. Per capita costs for selected interventions to prevent cardiovascular disease in LMIC settings compared to per capita expenditure on health for the year considered in that study. [file 1471-2458-13-285-S3.doc]

Appendix 3: Definitions used in this review

| **Terminology** | **Definition** | **source** |
| --- | --- | --- |
| Low income country | Gross National Income (GNI) per capita ≤ $995 | http://data.worldbank.org/about/country-classifications |
| Mid Income country | GNI per capita of $996 - $12,195. Mid income countries are sub-divided into lower middle income and upper middle income.  Lower middle income: $996-$3,945. Upper middle income: $3,946 -$12,195 | http://data.worldbank.org/about/country-classifications |
| High income | GNI per capita ≥ $12,196 | http://data.worldbank.org/about/country-classifications |
| Cardiovascular disease | Cardiovascular disease is defined here as all Hypertensive disease (ICD 10 code I10–I15), Ischaemic heart disease (ICD 10 code I20–I25), cerebrovascular disease (ICD 10 code I60–I69) and other cardiovascular diseases (ICD 10 code I26–I28, I34–I37, I44–I51, I70–I99) |  |
| Personal interventions | A broad term for any intervention applied at the level of the individual such as pharmacotherapy or pharmaco-prevention or individual behaviour or lifestyle modification. |  |
| Non personal intervention | A broad term for interventions applied at the group or population level, including interventions such as advertising and salt reduction in food. |  |
| High risk | No standard definition. Differing definitions used by authors of papers assessed here. |  |
